# Enhanced Surgical Techniques in Orthopedics: A Comprehensive Guide for Surgeons Based on Modification of the McFarland and Osborne Approach to the Hip

**DOI:** 10.7759/cureus.64711

**Published:** 2024-07-17

**Authors:** Chandra Shekhar, Anil K Joshi, Mohd R Warsi, Indrajit D Bhoumik

**Affiliations:** 1 Orthopaedics, Government Doon Medical College, Dehradun, IND

**Keywords:** modifications, post-operative mobility, bipolar hemiarthroplasty, total joint arthroplasty, trauma and orthopaedic surgery, orthopedic procedures

## Abstract

Introduction: The implementation of various approaches in hip arthroplasties introduces distinct advantages and complications. Notably, widely adopted methods such as the posterior approach have been linked to elevated rates of posterior hip dislocations and iatrogenic sciatic nerve injuries, while the lateral approach has been associated with superior gluteal nerve injuries.

In this study, we propose a refined modification of the McFarland and Osborne approach, aiming to amalgamate the most favorable aspects of prior modifications of the lateral approach to the hip. Additionally, our contribution extends to providing a comprehensive stepwise guide for the exposure and closure processes in cases of bipolar hemiarthroplasty or total hip replacement. This modification not only offers potential advantages to seasoned orthopedic surgeons but also serves as a valuable resource for young Turks venturing into hip surgeries.

Material and methods: 14 patients with femoral neck fractures underwent surgery using the modified McFarland and Osborne approach and were followed up for a period of six months. The functional outcome was analyzed by the Modified Mobility and Aids Scoring Matrix.

Results: Seven of the 14 patients attained pre-injury status with respect to the Mobility and Aids scoring matrix. six patients had a fall of 1, and one patient had a fall of 2, as compared to pre-injury status.

Conclusion: Our research suggests that this method serves as a superior alternative to conventional approaches, demonstrating notable advantages in terms of dissection difficulty, reduced risk to neurovascular structures, and minimized post-operative hip dislocations. Additionally, it exhibits a favorable outcome, enabling a return to pre-injury levels of activity.

## Introduction

Femoral neck fractures are common bony injuries that are often observed in elderly patients following trivial trauma [1]. Femoral neck fractures can also occur in young individuals following high-velocity trauma or polytrauma [1,2]. In the pediatric population, it is seen less frequently [3].

Accurate reduction and internal fixation are mandatory requirements to expect fracture healing in femoral neck fractures, as the hip is subjected to a very high degree of shearing strain due to the action of various groups of muscles acting on the hip [4,5].

Understanding the precise anatomical and biomechanical dynamics of the hip joint is crucial for orthopedic surgeons [6]. According to Al-Hayani [7], the Gluteus Medius and Gluteus Minimus each consist of multiple distinct parts, with separate branches from the superior gluteal nerve serving each part. These muscles play a critical role in stabilizing the femoral head within the acetabulum throughout various stages of the gait cycle. Specifically, the posterior segments of the Gluteus Medius and Minimus, aligned parallel to the femoral neck, contribute significantly to this stability. Furthermore, the Tensor fasciae latae aids in stabilizing body weight and the hip joint, owing to its alignment parallel to both the body's weight line and the femoral axis.

The treatment of displaced femoral fractures has changed over the years from reduction and internal fixation to arthroplasty of the hip joint. Hemiarthroplasty is done using different approaches. The hip joint can be approached using either of the two approaches [8]. 1) Approaching the hip joint with an intact Gluteus Medius (Hueter’s [9] and Langenbeck’s [10] approach) 2) Approaching the hip joint by dividing the continuity of the Gluteus Medius (Smith-Petersen [11]) or by dividing its insertion (Kocher, Murphy, Ollier, Brackett [12]). In 1954, Bryan McFarland and Geoffrey Osborne described a method of preserving the integrity of the Gluteus Medius muscle during the posterior approach to the hip joint.

In 1984, a direct lateral approach to the hip via the Gluteus Medius was described by James McLauchlan [13]. This approach builds upon anatomical observations made by McFarland and Osborne in 1954, who noted that the Gluteus Medius and the Vastus Lateralis are functionally continuous through the thick periosteum covering the greater trochanter.

In 1986, Desmond Dal described osteotomy of the anterior part of the greater trochanter with retention of attachments of the Gluteus Medius, Vastus Lateralis, and Gluteus Minimus to it [14].

The purpose of this study is to provide a stepwise approach for bipolar hemiarthroplasty in patients with neck or femur fractures using the principles of Bryan McFarland and Geoffrey Osborne. This study will also focus on a minimally invasive approach and reattachment of the periosteal sleeve to the greater trochanter.

## Materials and methods

The study includes patients who underwent hemiarthroplasty of the hip at Government Doon Medical College and Hospital, Dehradun. 14 patients with femoral neck fractures underwent surgery using the modified McFarland and Osborne approach and were followed up for a period of 6 months in the outpatient department. The functional outcome was analyzed by the Modified Mobility and Aids Scoring Matrix [15].

Operative technique

A modified version of the lateral approach to the hip given by Bryan McFarland and Geoffrey Osborne is employed. After anesthesia, the patient is positioned in the lateral decubitus position, and standard protocol is followed for preparing the surgical site with an antiseptic solution and draping it. An antimicrobial incise drape is finally applied to the incision area.

**Figure 1:**
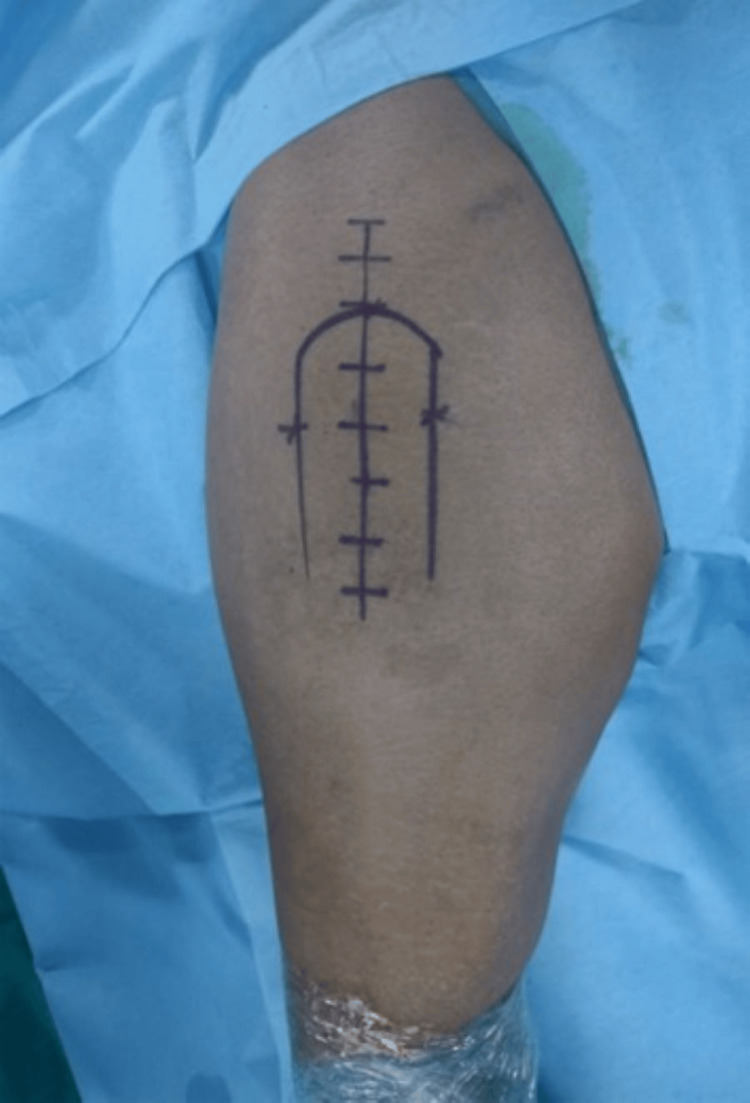
Marking the incision.

The hip is kept flexed at 15 degrees, and a straight skin incision of around 12-15cm is made centered on the greater trochanter (Figure 1). The incision was around 2cm proximal to the tip of the trochanter, extending approximately 10 cm distally.

**Figure 2:**
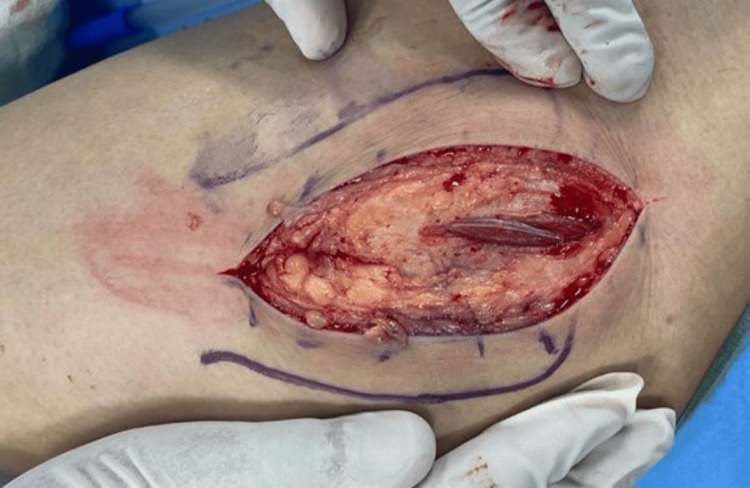
The fat and fascia lata are incised in line with the skin incision.

The length of the incision can be extended distally, depending on each patient. The fat and fascia lata were incised along the line of the skin incision (Figure 2) and retracted using a self-retaining retractor. Proximally, the gluteus maximus insertion into the fascia lata was identified and split along the same line as the fascia lata.

**Figure 3:**
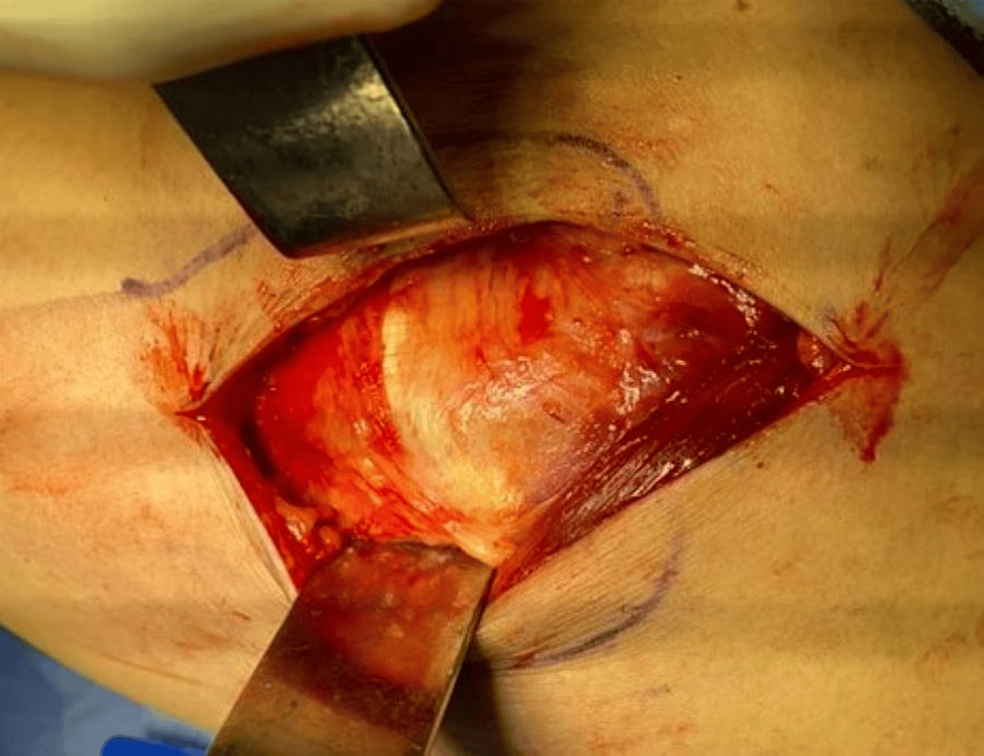
Trochanteric bursa.

**Figure 4:**
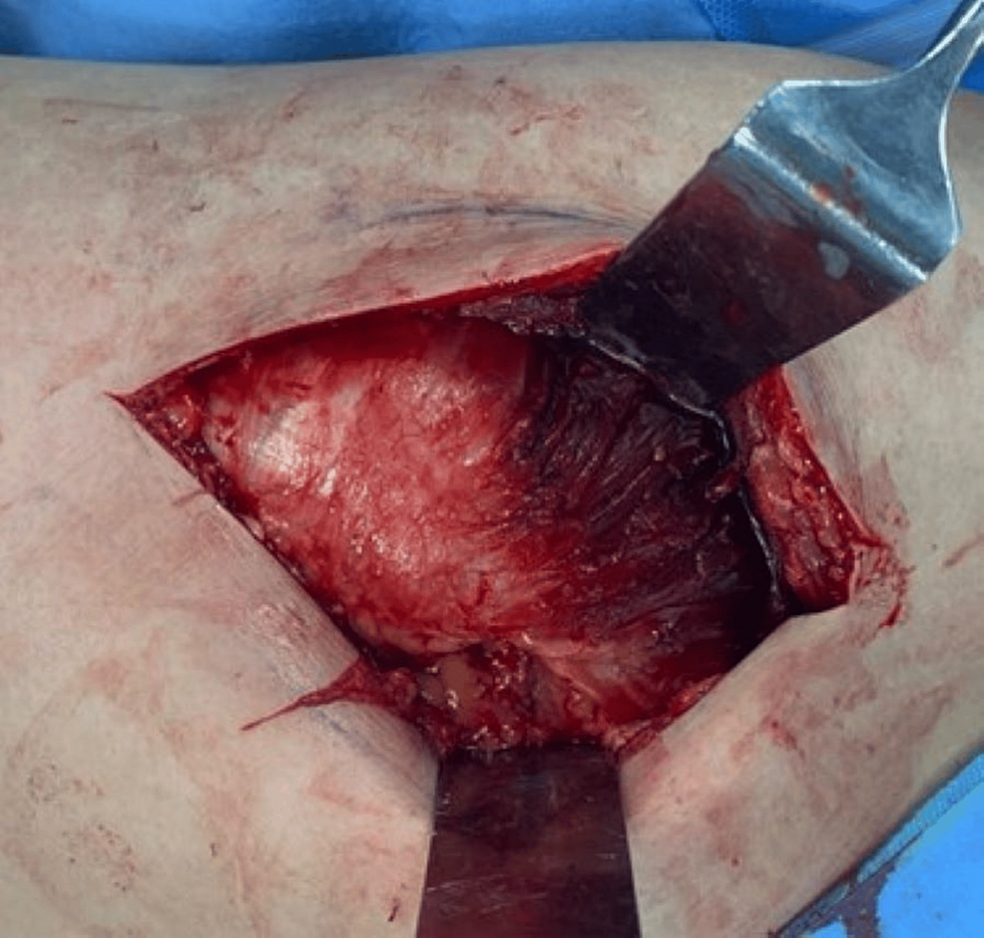
The anterior and posterior borders of the gluteus medius and vastus lateralis.

The trochanteric bursa (Figure 3) was incised to demonstrate the anterior and posterior borders of the gluteus medius and vastus lateralis (Figure 4).

**Figure 5:**
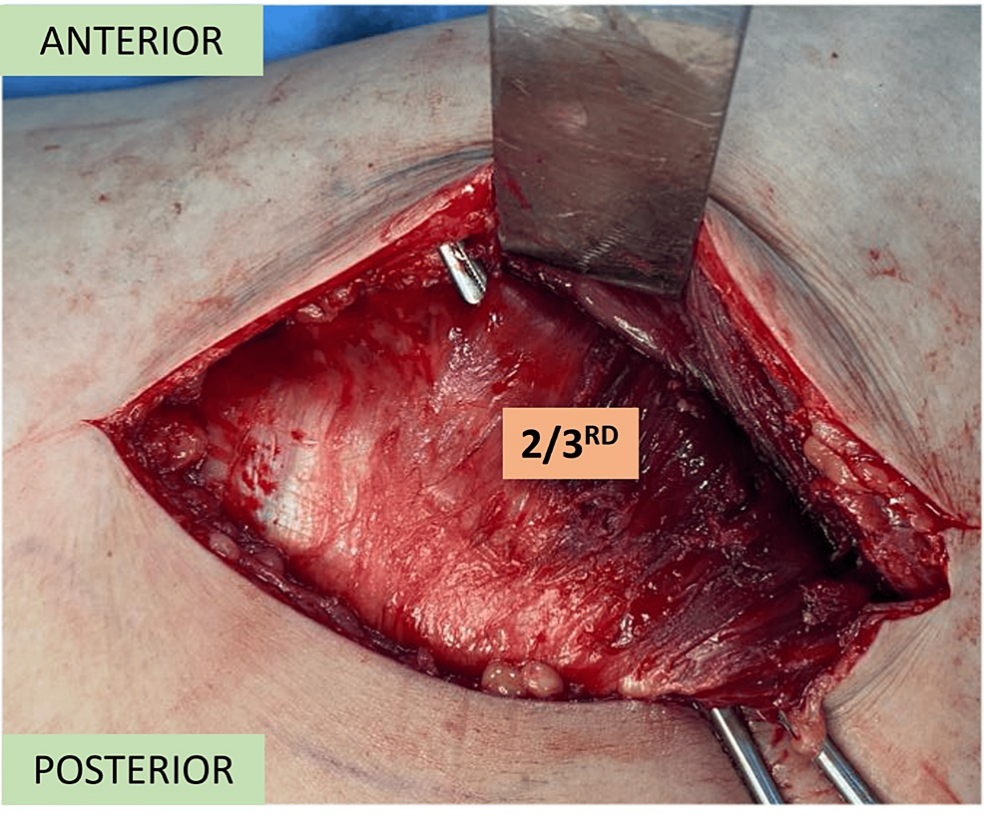
Anterior 2/3rd of the gluteus medius.

Blunt dissection is used to split the anterior 2/3rd of the gluteus medius (Figure 5), which generally makes an anterior 75-degree angulation to the skin incision.

**Figure 6:**
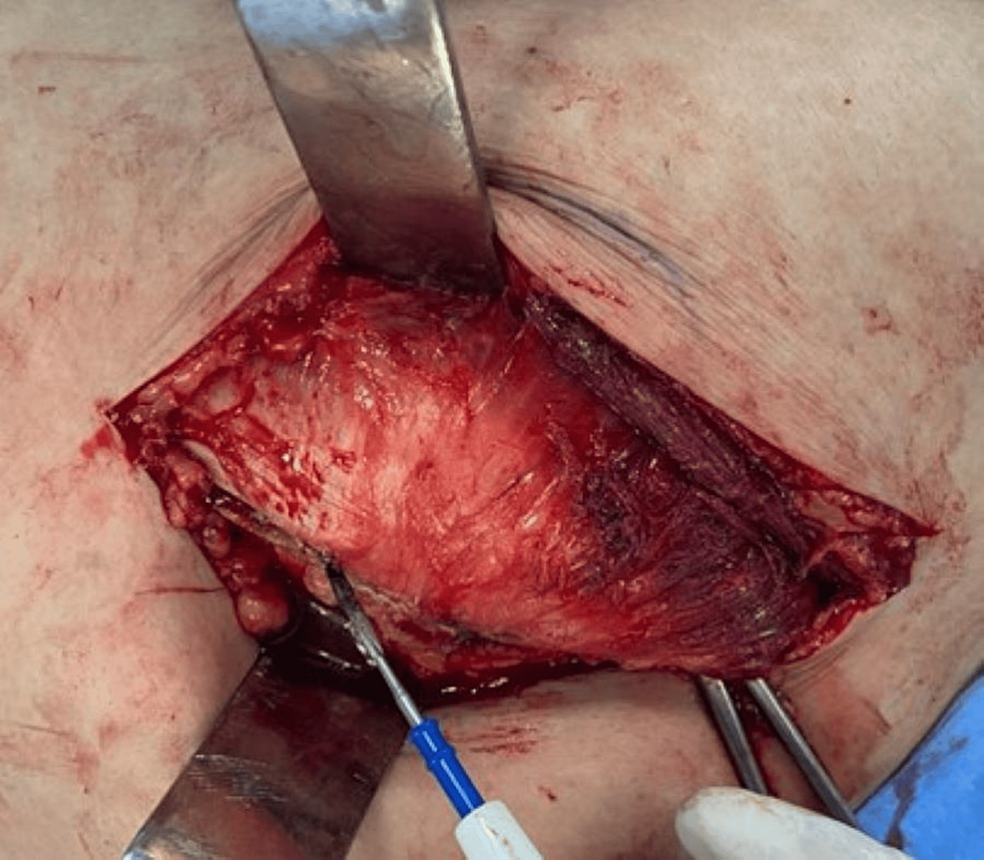
Blunt dissection through the anterior 2/3rd part of the vastus lateralis.

**Figure 7:**
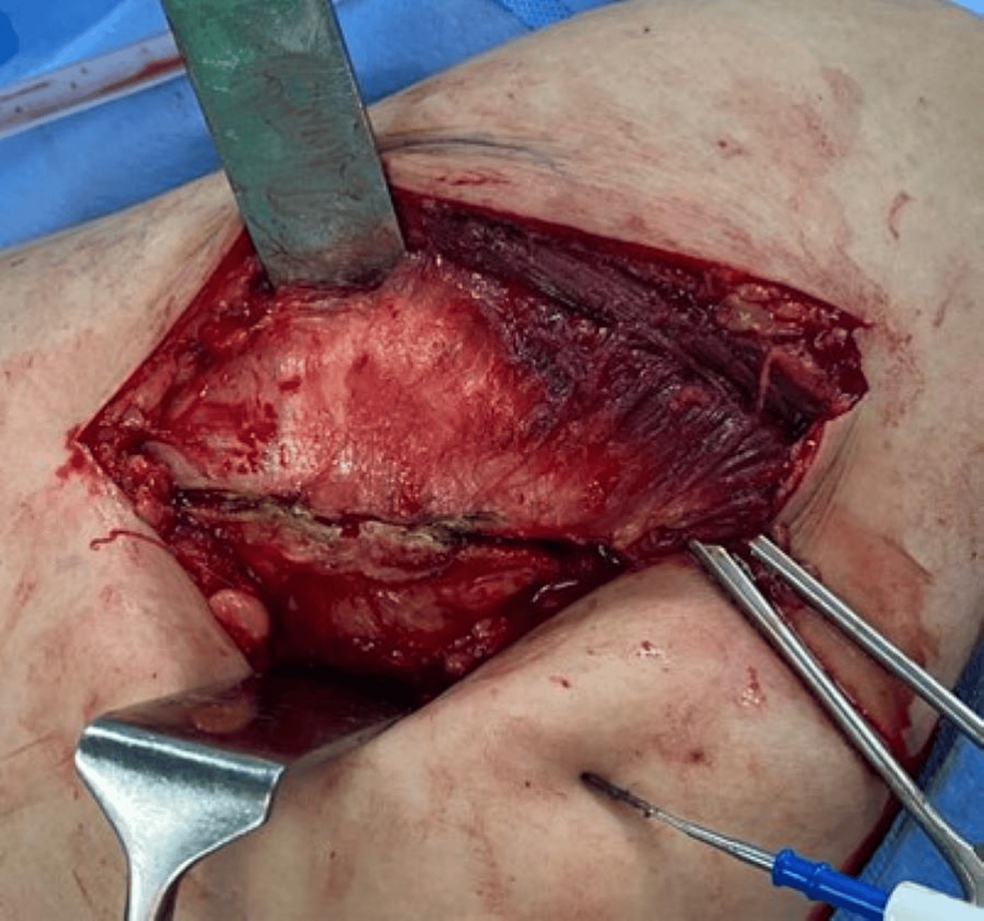
Blunt dissection of vastus lateralis completed.

The split is not extended more than 3cm cephalad to protect the superior gluteal nerve. Next, distal blunt dissection was carried out through the anterior 2/3rd part of the vastus lateralis passing down to the bone for about 3 cm (Figures 6, 7).

**Figure 8:**
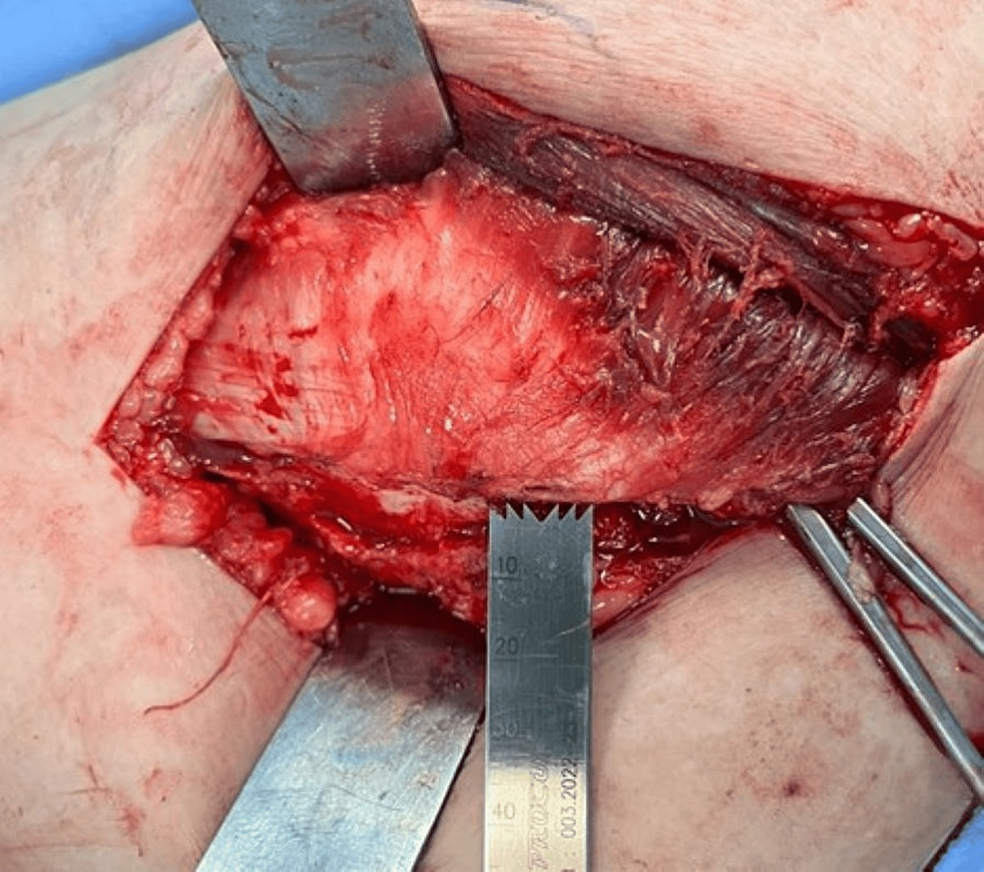
Power saw for osteoperiosteal sleeve.

**Figure 9:**
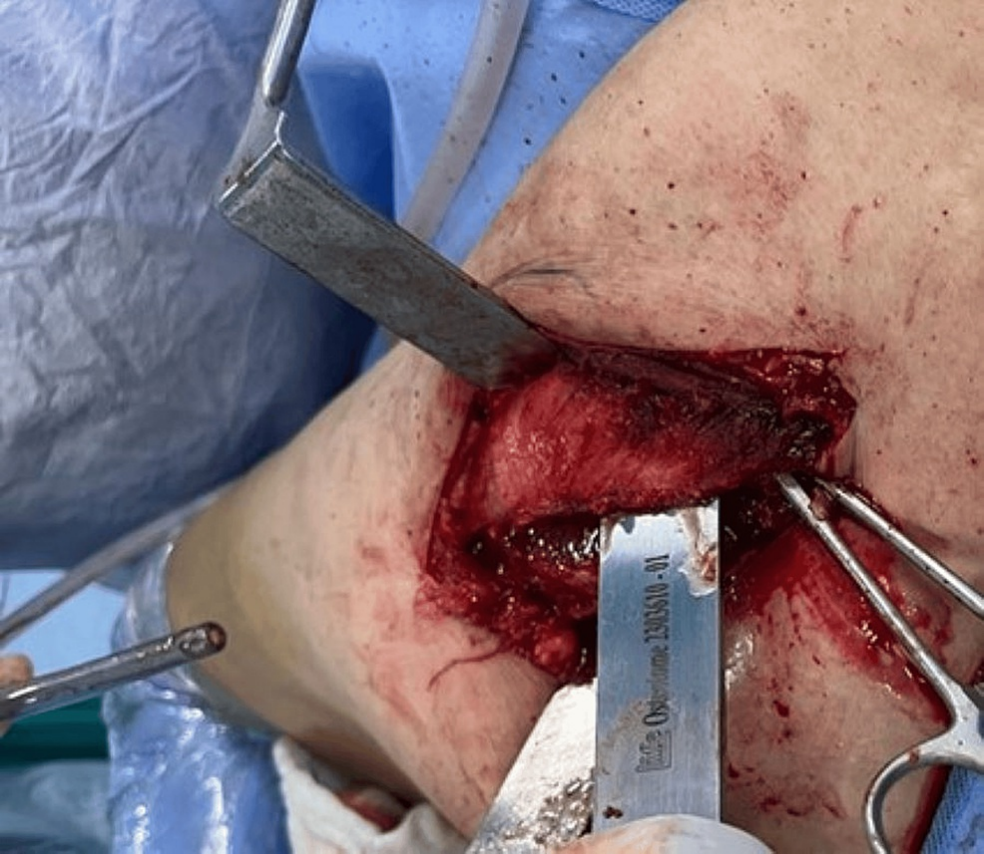
An osteoperiosteal sleeve of the greater trochanter.

**Figure 10:**
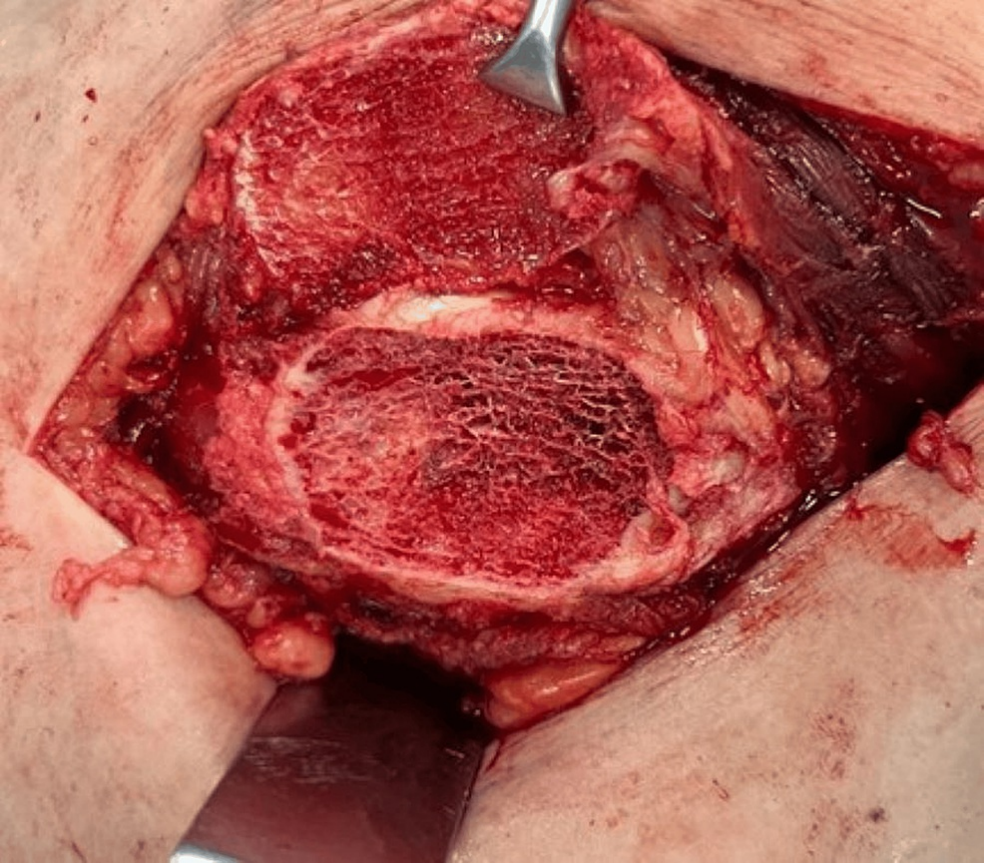
Osteoperiosteal sleeve.

An osteoperiosteal sleeve of the greater trochanter of around 3-5mm thickness is then made (Figures 8, 9) using a power saw with the gluteus medius attached to it proximally and the vastus lateralis distally, and this entire cuff is reflected anteriorly (Figure 10).

**Figure 11:**
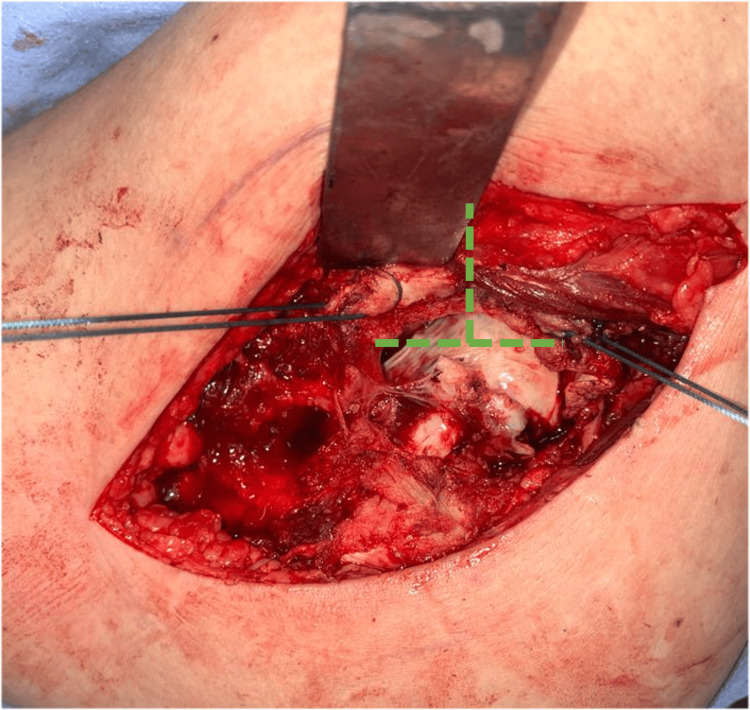
Gluteus minimus and anterior capsule are tagged and cut using diathermy.

The limb is then rotated externally. Gluteus minimus and anterior capsule are cut together using diathermy with a ‘t’ shaped incision (Figure 11) and are tagged using nonabsorbable polyester sutures.

**Figure 12:**
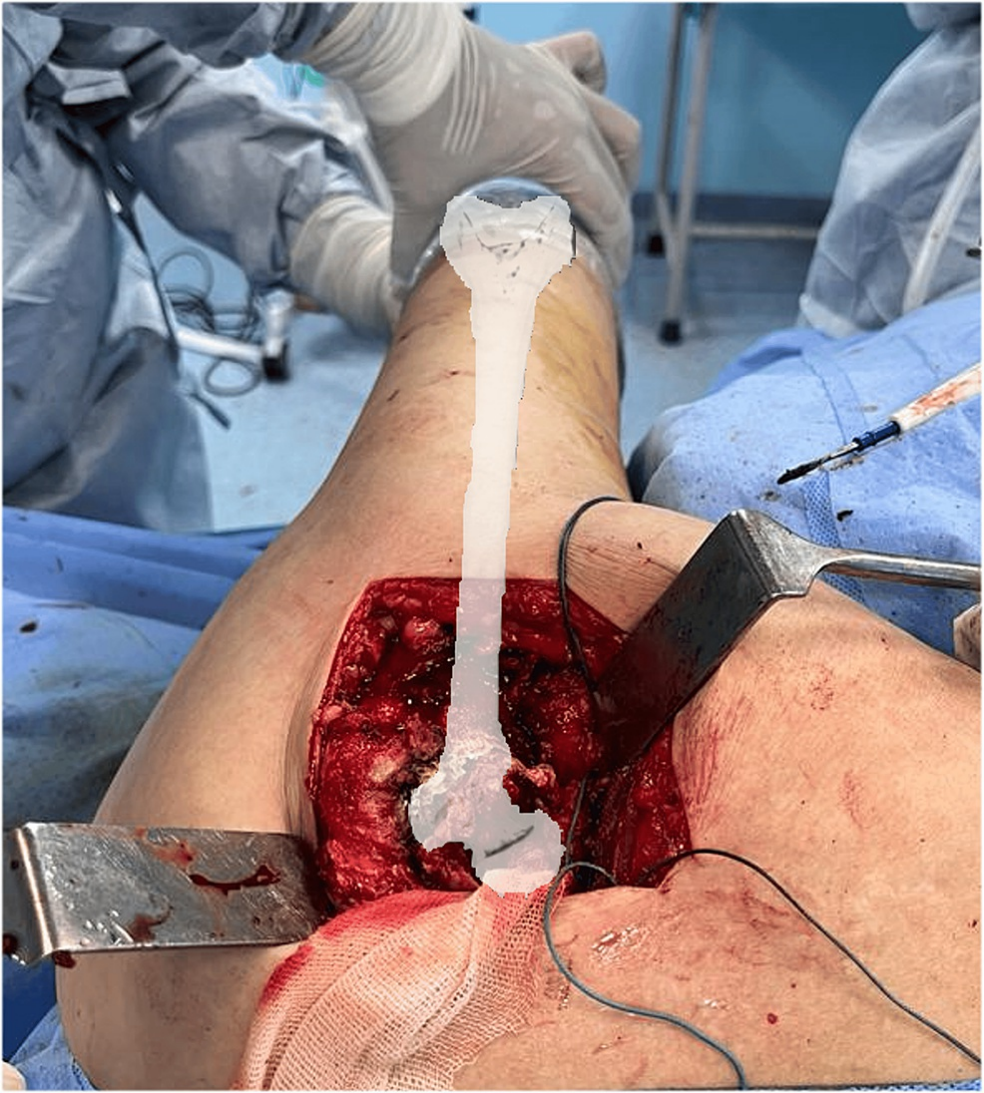
The comprehension of the version of the femoral neck.

**Figure 13:**
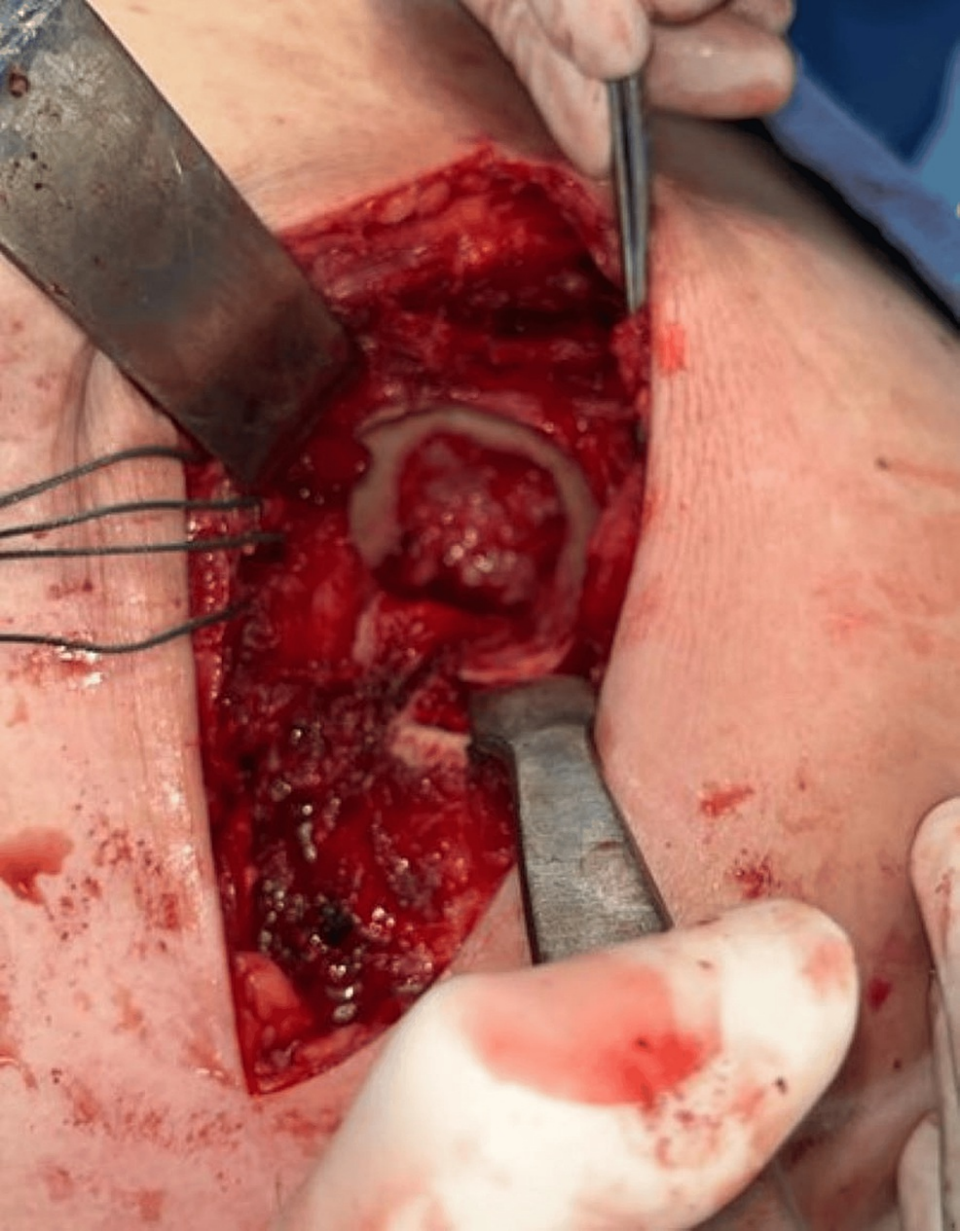
Visualising acetabulum.

The femoral head is then removed, and two pin retractors are used to better visualize the acetabulum (Figure 13). The hip is flexed 90 degrees and as the leg falls forward on the far side of the surgeon this makes the anterior surface of the proximal femur come under direct visualization of the operating surgeon which makes the comprehension of the version of the femoral neck easier (Figure 12). 

**Figure 14:**
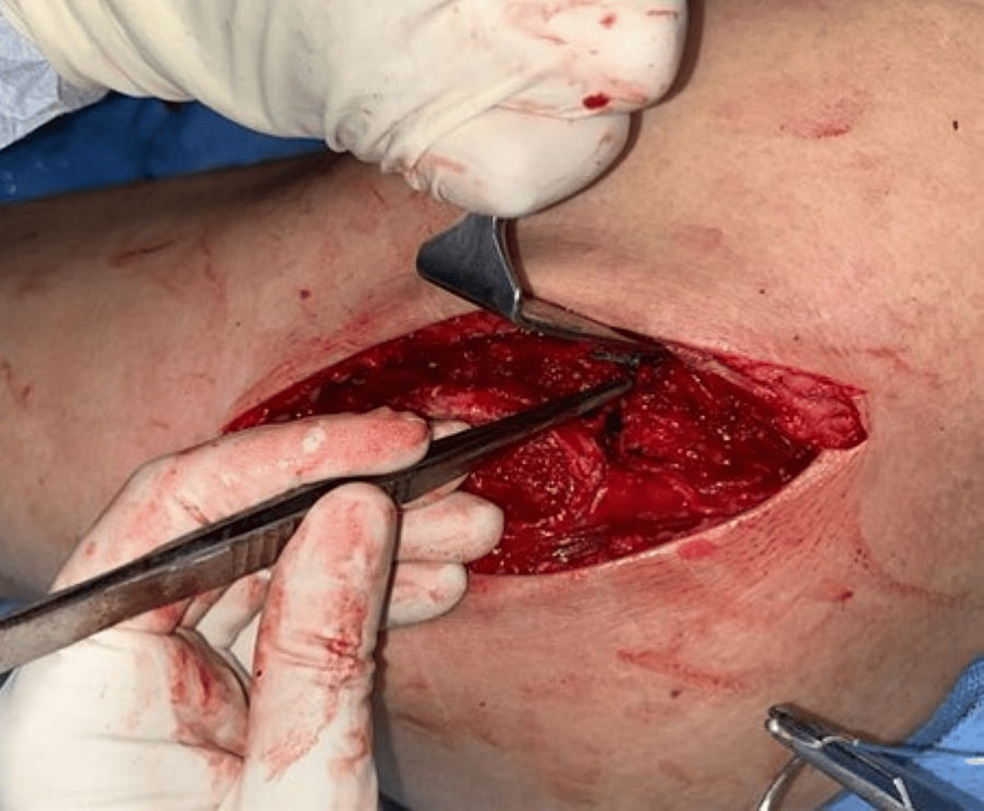
Gluteus minimus and anterior capsule are sutured together.

During closure, the tagged gluteus minimus and anterior capsule are sutured together (Figure 14) to make the capsule tenacious.

**Figure 15:**
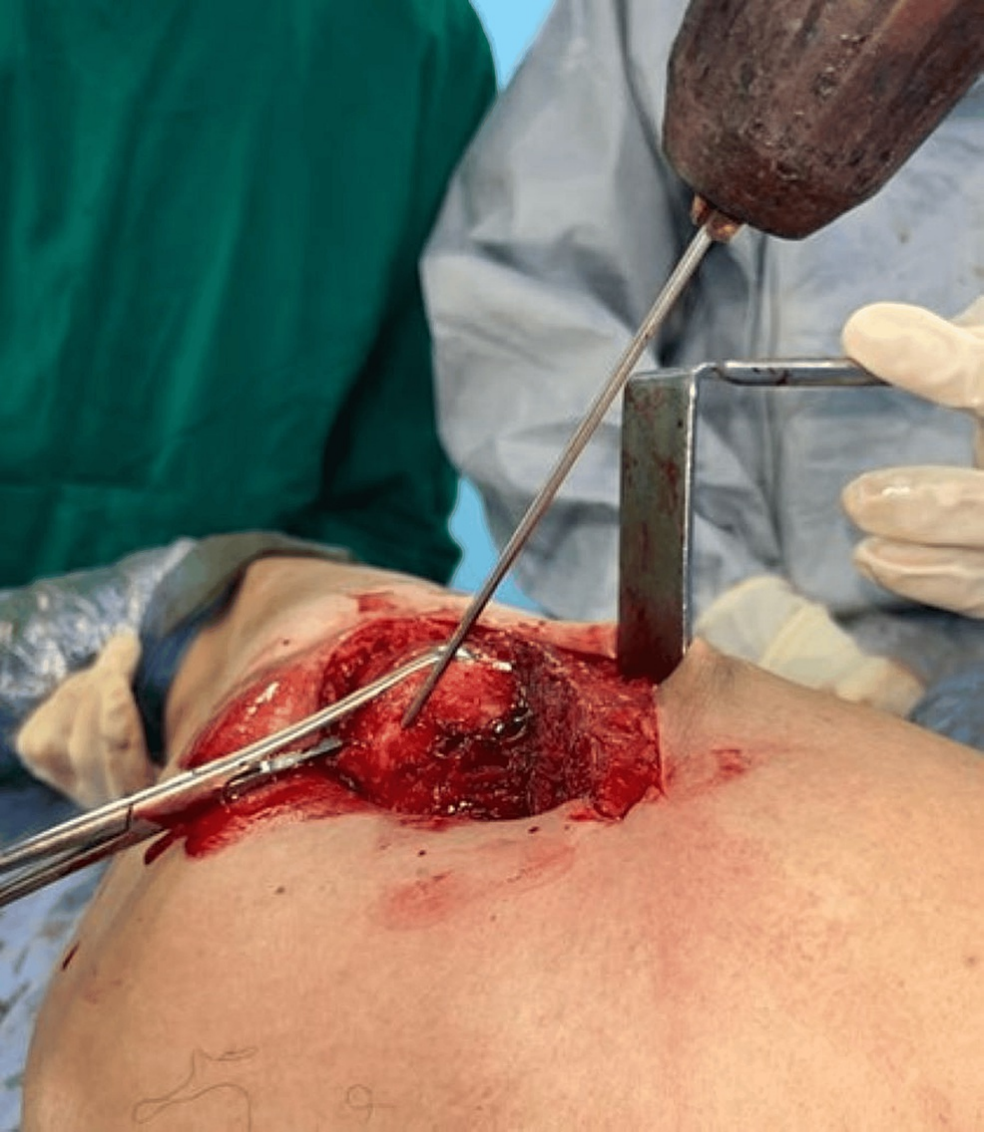
Drilling four holes from superior to posterior surface of the greater trochanter.

**Figure 16:**
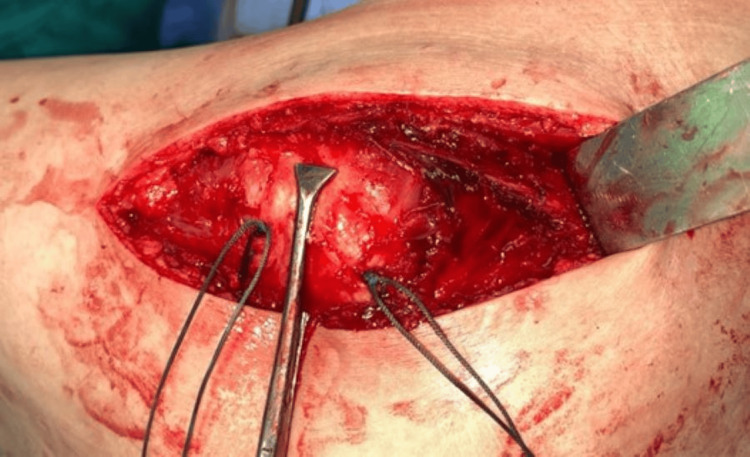
Securing osteoperiosteal sleeve.

The osteoperiosteal sleeve is attached back to the greater trochanter using four strands of the non-absorbable polyester sutures, one proximally and the other distally. This is done by drilling four holes from the superior to the posterior surface of the greater trochanter and then passing the sutures (Figures 15, 16).

**Figure 17:**
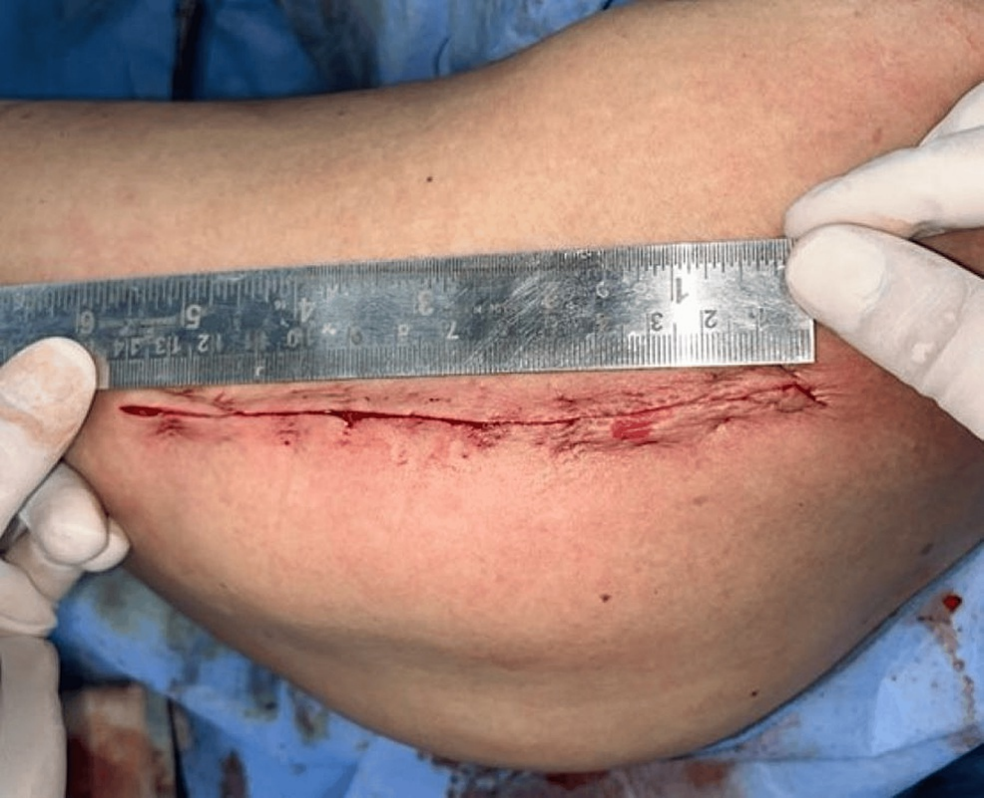
Closure.

Meanwhile, the limb is kept in slight abduction to facilitate in proper positioning of the osteoperiosteal sleeve. The fascia lata and subcutaneous tissues are closed with non-absorbable vicryl sutures and the skin is closed with staples or nylon (Figure 17).

## Results

Fourteen patients with femoral neck fractures underwent surgery using the modified McFarland and Osborne approach and were followed up for six months. The study comprised nine females and five males, with an average age of 62 years. Most patients (13 out of 14) presented within 1 to 2 weeks of injury, except for one patient who presented after three weeks.

Among the surgeries, six of the fourteen hemiarthroplasties were cemented, thirteen were modular and one was fixed. Notably, there were no intra-operative neurovascular injuries, fractures, post-operative hip dislocations, or wound infections reported.

**Figure 18:**
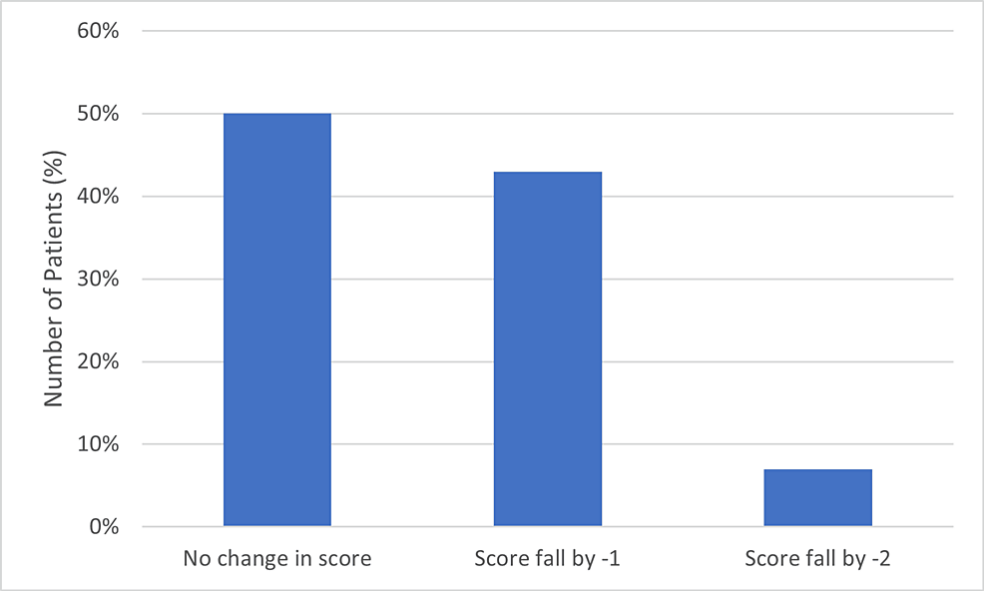
Mobility and aid score at one month.

By the end of the first month, 7 out of 14 patients had regained their pre-injury status according to the Mobility and Aids scoring matrix. Six patients had a decrease in their score by 1 point, and one patient had a decrease of 2 points compared to their pre-injury status (Table 1, Figure 18).

**Table 1:** Mobility and aids scoring after one month of follow-up.

Mobility and Aid Score Before Surgery (N = Number of Patients)	Mobility and Aid Score after Surgery (Bipolar Hemiarthroplasty)	
	Change in Score	No. of patients
Score of 7 (N=6)	No Change	3
	fall by -1	2
	fall by -2	1
Score of 6 (N=5)	No Change	3
	fall by -1	2
	fall by -2	0
Score of 5 (N=2)	No Change	1
	fall by -1	1
	fall by -2	0
Score of 4 (N=1)	No Change	0
	fall by -1	1
	fall by -2	0

**Figure 19:**
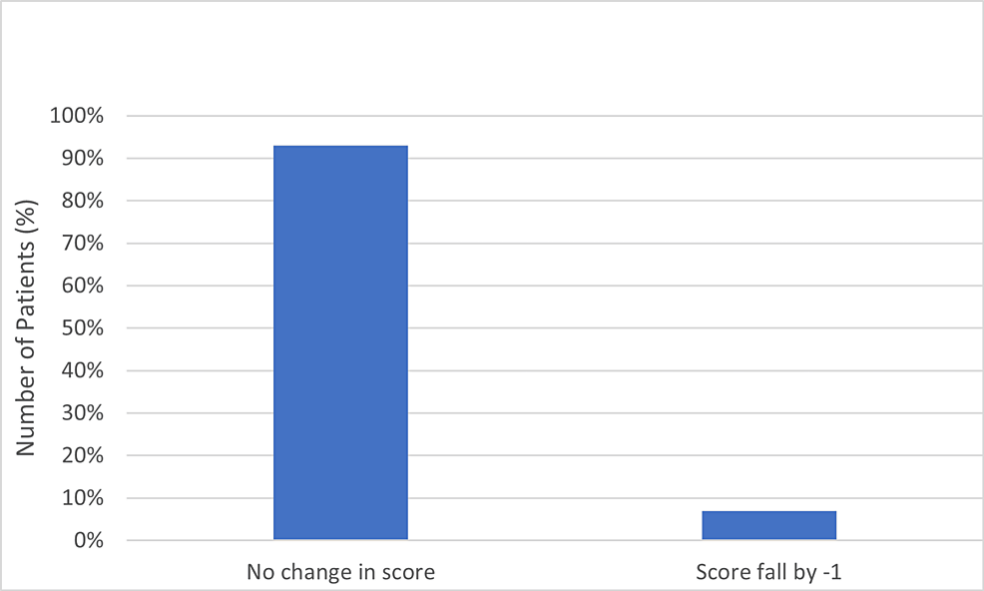
Mobility and aid score at three months.

The modified McFarland and Osborne approach shows promising results in the follow-up period with 13 out of the 14 cases having a return of pre-injury function within 1 to 3 months according to the modified mobility and aids scoring matrix (Table 2, Figure 19).

**Table 2:** Mobility and Aids score after three months of follow-up.

Mobility and Aid Score Before Surgery (N = Number of Patients)	Mobility and Aid Score after Surgery (Bipolar Hemiarthroplasty)	
	Change in Score	No. of patients
Score of 7 (N=6)	No Change	6
	fall by -1	0
	fall by -2	0
Score of 6 (N=5)	No Change	4
	fall by -1	1
	fall by -2	0
Score of 5 (N=2)	No Change	2
	fall by -1	0
	fall by -2	0
Score of 4 (N=1)	No Change	1
	fall by -1	0
	fall by -2	0

**Figure 20:**
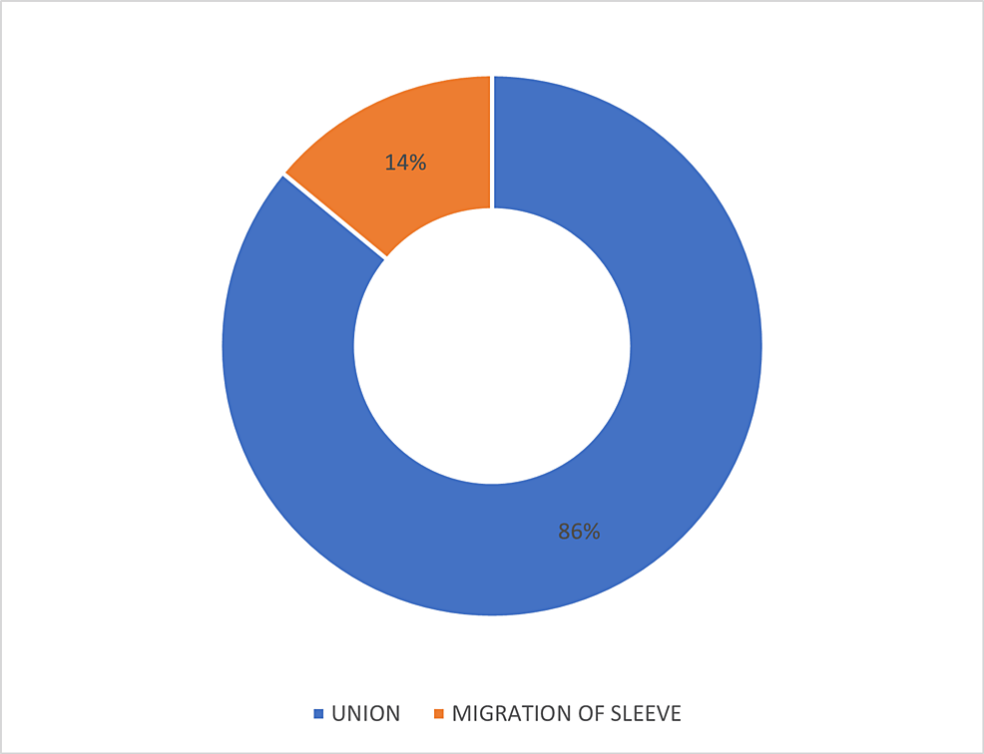
Osteoperiosteal sleeve migration.

A bony union of the osteoperiosteal sleeve is seen in 12 patients, and migration of the sleeve by around 1 cm is seen in two patients (Figure 20).

## Discussion

Bipolar hemiarthroplasty is a well-established procedure in patients with fractures of the neck of the femur [16,17]. This surgery has undergone considerable evolution over the years since its advent. Despite these modifications, orthopedic surgeons still encounter intraoperative struggles and postoperative complications. There is always reluctance among surgeons to change their conventional surgical approach.

The modified McFarland and Osborne approach to the hip is used with good outcomes in the patients under review. A range of complications, like postoperative hip dislocations, neurovascular injuries, and heterotopic ossification, were not encountered during the course of this study. This study uses the modified mobility and aids scoring matrix for evaluating the outcome in patients undergoing bipolar hemiarthroplasty. Using this scoring matrix, we compared the pre-injury level of function and the post-operative outcome. Compared to the Harris hip score, which only addresses the outcome of surgery, this scoring system offers a comparative approach, especially in patients who already had restricted mobility before sustaining injury.

The modified approach provides easy access to the hip joint and excellent exposure to both the acetabular and proximal femoral regions. In this modified approach, the posterior capsule is intact and the anterior capsule is repaired, providing a soft tissue envelope around the joint. Dislocation occurs infrequently, and postoperative migration of the sleeve is not a major problem. Heterotopic ossification is a known complication after hip arthroplasty [18], though not encountered during our study, can be dealt with prophylactic Indomethacin as per the known literature [19]. 

The aim of this study is to provide a stepwise and comprehensive approach to a young orthopedic surgeon with minimal expertise in arthroplasty surgeries. The study also focuses on reducing post-operative complications and ensuring an easy return to function.

Despite the study's limitations, including a small patient sample, lack of a comparative group, absence of data on total hip arthroplasty, and an average follow-up period of six months, the findings suggest that this approach is promising and warrants consideration as a valid and encouraging option for bipolar hemiarthroplasty. It holds the potential to deliver improved outcomes to patients undergoing this procedure.

## Conclusions

Our research suggests that this method serves as a suitable alternative to conventional approaches, demonstrating notable advantages such as easier dissection, reduced neurovascular injuries, minimal post-operative hip dislocations, and a return to pre-injury levels of activity.
